# Large Gluteal Cystic Lesion in a Child: A Challenging Diagnosis of a Hydatid Cyst

**DOI:** 10.7759/cureus.48958

**Published:** 2023-11-17

**Authors:** Mohammad Badr Almoshantaf, Rami Soliman, Manar Abdullah, Hala bastati, Shorouk Alabdo, Azza Bakr Ahmed, Wael Hafez

**Affiliations:** 1 Neurosurgery, Ibn Al-Walid Hospital, Homs, SYR; 2 Pulmonology/Respiratory, National Center for Allergy and Chest Diseases, Cairo, EGY; 3 Pediatric Surgery, Aleppo University Hospital, Aleppo, SYR; 4 General Medicine, Ain Shams General Hospital, Cairo, EGY; 5 Internal Medicine, The National Research Center, Cairo, EGY; 6 Internal Medicine, NMC Royal Hospital, Abu Dhabi, ARE

**Keywords:** weinberg, gluteal, hydatid cysts, child, albendazole

## Abstract

Early identification of asymptomatic hydatid cysts, produced by Echinococcus parasites in their larval form, is crucial due to their frequent late-stage diagnosis. Radiological imaging plays a significant role in early detection. These cysts mostly impact the liver and lungs but may manifest in other areas of the body, presenting distinct diagnostic difficulties. This case example emphasizes the need for using radiological imaging and maintaining a high level of suspicion when it comes to identifying hydatid cysts in young patients. We present an exceptional case of a three-year-old child in good health who developed a painless gluteal enlargement that resulted in constipation. An abdominal CT scan detected a hydatid cyst located posterior to the bladder, along with an accompanying hepatic cyst. The diagnosis was confirmed by a positive Weinberg test, and the treatment consisted of a 28-day course of albendazole. The key takeaway from this case report is that prompt diagnosis and radiological imaging play a critical role in instances of hydatid cysts.

## Introduction

Echinococcus granulosus, a zoonotic illness caused by tapeworm larvae belonging to the genus Echinococcus, is a global phenomenon. In humans, who serve as intermediate hosts, these larvae have the ability to migrate via the circulatory or lymphatic systems and mature in different anatomical sites [1]. Hydatid cysts, which are a symptom of this illness, are seldom seen in the pelvic area. Usually, these cysts occur when a hepatic hydatid cyst ruptures on its own [2]. Manifestations are typically lacking, and infection is commonly identified fortuitously by imaging investigations. Hydatid cysts mostly impact the liver and lungs, although they may also occur in unusual sites like the spleen, kidney, muscles, brain, spine, breast, thyroid, peritoneal cavity, and retroperitoneum. The kidneys are the organs most often afflicted in the urinary system, whereas involvement of the urinary bladder is quite uncommon [3,4]. Ultrasonography is essential for diagnosis, but computed tomography (CT) scans provide significant information when the diagnosis is still unclear. In this report, we describe an uncommon occurrence of a hydatid cyst located in the gluteal region, which exerted pressure on the anal canal and adjacent pelvic areas in a young patient, resulting in constipation. This instance posed a complex diagnostic challenge and introduced a distinctive element to the clinical situation. 

## Case presentation

A medically fit three-year-old youngster was admitted to the pediatric department at Aleppo University. The infant's medical history, family history, drug history, and allergy history were all normal for a newborn born via Cesarean section. The toddler experienced a painless episode of constipation lasting for two days which brought her to the emergency department. During the medical examination, the child's parents reported the absence of any signs of systemic fever, bloody discharge, history of insect bites, or contact with animals. The physical examination did not reveal any specific findings. The levels of C-reactive protein (CRP), leukocytes, and lactate were all within the normal range. Based on the lack of findings, a request was made for an ultrasound examination. The ultrasound assessment detected a well-defined cystic structure positioned posterior to the bladder. The dimensions of this cyst were around 5x3 cm, and it extended into the right gluteal region.

**Figure 1:**
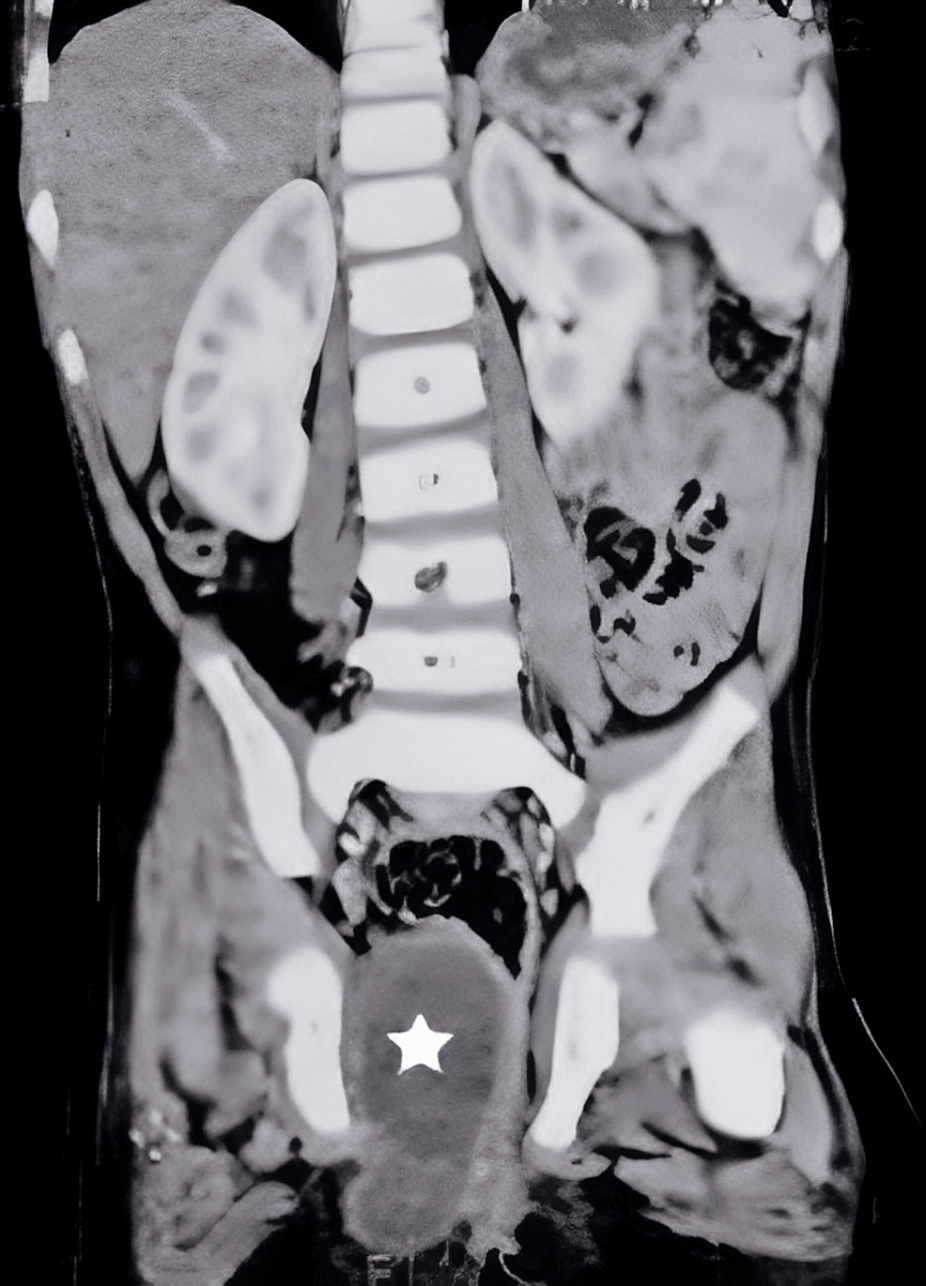
CT showing a 5x3 cm cystic lesion (asterisk) in the gluteal region compressing the anal canal.

**Figure 2:**
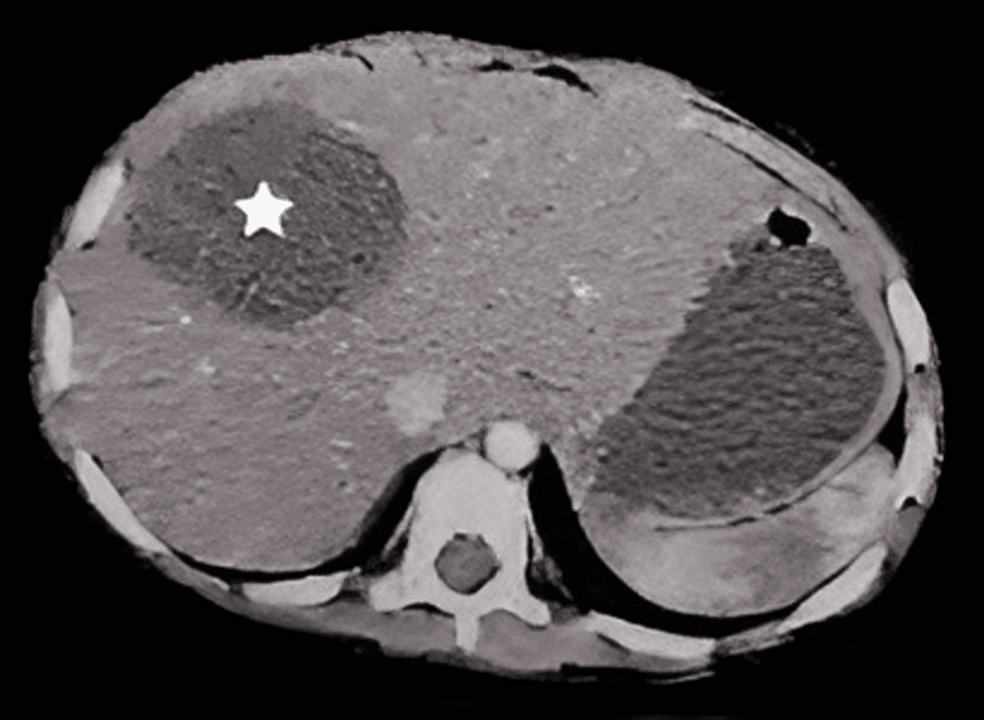
CT showing a 4x2 cm cystic lesion (asterisk) in the liver.

Subsequently, a CT scan revealed indications of a hydatid cyst (Figure 1). In addition, the CT scan exposed the existence of an extra cyst measuring 4x2 cm in the liver, which further complicated the diagnostic situation (Figure 2). The Weinberg test was performed and produced a positive outcome, with a measurement of 28.7 U. After being diagnosed, the patient began a treatment regimen that included taking albendazole orally for a duration of 28 days. Rectal glycerin suppositories alleviated the constipation. 

## Discussion

Hydatid disease can remain asymptomatic for an extended period of time. However, as hydatid cysts develop in your liver, lungs, or other organs, they might result in various symptoms. In this case, the hydatid cyst remained without symptoms until it had enlarged substantially, resulting in constipation. The cyst grew to a significant size and began to constrict the anal canal. The delay in the child’s hospital admission by the parents was potentially influenced by poor sociocultural factors. Upon imaging, the cyst was observed as a palpable lump with solid components, displaying tightness and partial firmness in the gluteal region. Although the serological tests used in this case are not exceptionally reliable, they are clinically significant for monitoring patients for possible relapse [5]. 

During the diagnostic procedure, medical professionals frequently utilize radiological methods such as ultrasonography and CT. In this case report, CT scans offered a more extensive range of information for evaluation of the hydatid cyst, identification of possible complications, and assisting in differential diagnosis, in comparison to ultrasound. Diagnostic criteria for hydatid cysts include radiological features such as a thick cyst wall, the presence of a germinative membrane, daughter cysts, and calcifications on the wall. However, differentiation of hydatid cysts from other uncomplicated cysts can be difficult, especially in the early stages when hydatid cysts appear as cysts without echoes, containing transparent fluid with thin walls. 

The main objective of treatment is to extract the fluid and cavities of the cyst to prevent contamination. Various therapeutic modalities are currently accessible, such as pharmacological interventional drugs like albendazole and mebendazole, endoscopic treatments such as Puncture, Aspiration, Injection, and Respiration (PAIR), as well as open surgery. PAIR is typically not the favored option for pediatric patients due to concerns over sedation, contamination, and the possible danger of allergy caused by contamination. However, PAIR may be considered for individuals who do not show improvement with medical treatment, have recurring episodes, or are not eligible for surgery [6,7]. 

Endoscopic operations are frequently avoided, particularly for pediatric patients, due to concerns regarding possible contamination [8]. Arroud et al. showed a pediatric case that exhibits both similarities and differences in comparison to ours where it involves a 12-year-old female patient [9]. Both cases were pediatric patients who presented with detectable abdominal tumors, which posed diagnostic difficulties. CT scans were essential in diagnosis of the condition. Nevertheless, there were significant variations in the anatomical positions and features of the cystic forms. The presented case involved a three-year-old child with a cystic formation located posterior to the bladder and an unforeseen cyst on the liver. On the other hand, in the previously reported case, a fluid-filled growth situated in the urachal region of the front part of the abdomen was present, which ultimately necessitated surgery to remove it [10]. Both cases emphasize the significance of medical treatment after diagnosis, specifically using albendazole (7.5 mg/kg/dose twice daily for six months) in our case and postoperative albendazole in the previously published case. These cases also highlight the various clinical presentations and management approaches for pediatric patients with cystic lesions in the pelvic region. 

## Conclusions

This case involving cystic abdominal lesions in a young patient highlights the intricate diagnostic challenges and distinctive clinical manifestations of cysts observed in children. Radiological examinations and serological assays were pivotal in directing the diagnosis, resulting in efficacious therapy. This case study expands our understanding of gluteal cystic lesions in children and underscores the importance of pursuing a wide range of diagnostic options. 
